# Preoperative endoscopic tattooing for colonic polyp localization: From blue to black

**DOI:** 10.1002/ccr3.2428

**Published:** 2019-09-27

**Authors:** Konstantinos Blouhos, Konstantinos A. Boulas, Aikaterini Paraskeva, Ioanna Gravalidou, Konstantinos Chatzipourganis, Alexandros Triantafyllidis, Anestis Hatzigeorgiadis

**Affiliations:** ^1^ Department of General Surgery General Hospital of Drama Drama Greece

**Keywords:** colonoscopy, tattooing, methylene blue, surgery, polypectomy, perforation

## Abstract

When surgical polypectomy and not segmental resection is planned, preoperative endoscopic tattooing with high‐volume undiluted methylene blue should be avoided as it can result in colon perforation.

## Graphical abstract

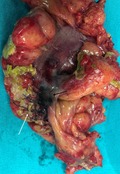

A 76‐year‐old male patient referred to our surgical endoscopy department for polypectomy of a large pedunculated distal sigmoid villous adenoma. As the polyp considered endoscopically unresectable, tattooing with 2 mL four‐quadrant submucosal injection of undiluted methylene blue (10‐mL sterile vials, 0.5%‐50 mg/10 mL) was performed. Immediate open polypectomy with minor colotomy was performed instead of segmental resection. On postoperative day 3, symptoms and signs of peritonitis developed. CT depicted rectosigmoid wall thickening and pelvic gas and fluid extravasation (Figure 1). Relaparotomy revealed blue discoloration of the colotomy site along with patchy black serosal areas and suture line rupture treated with Hartmann's procedure (Figure 2). Histology revealed transmural sigmoid wall necrosis at the colotomy site.

**Figure 1:**
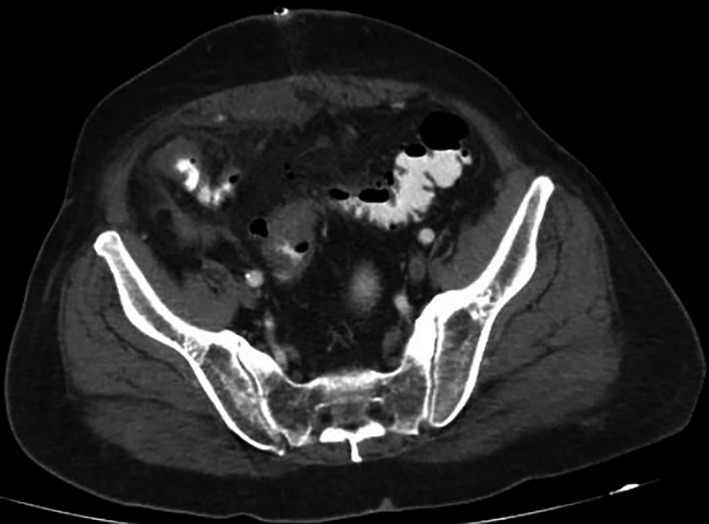
CT depicted extensive rectosigmoid wall thickening and pelvic extravasation of gas and fluid

**Figure 2:**
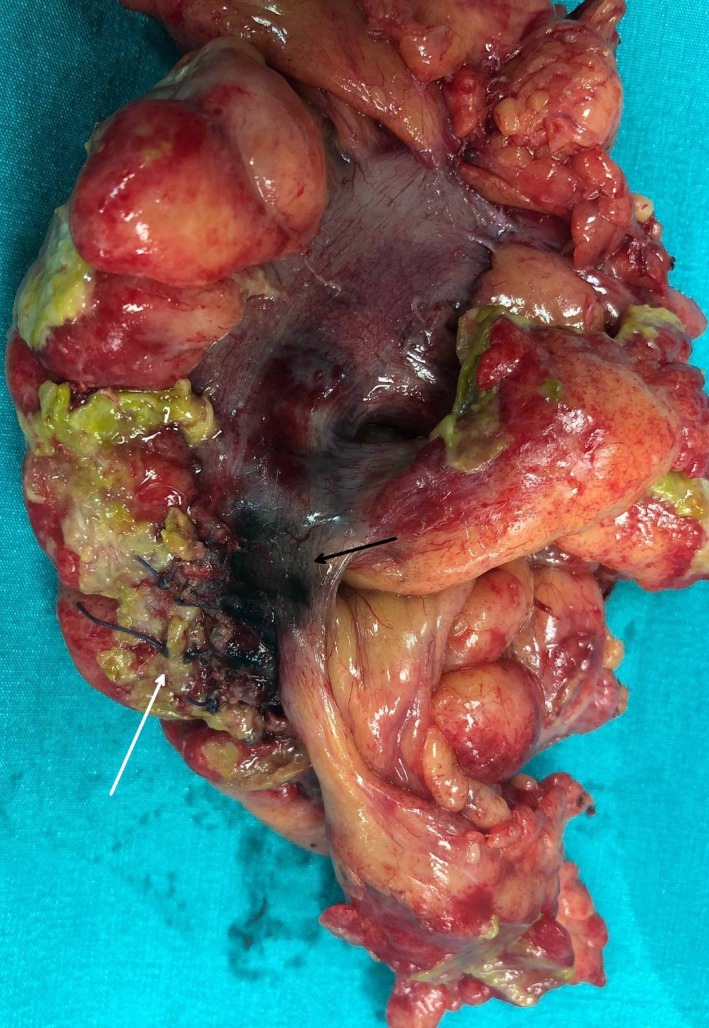
Relaparotomy revealed rectosigmoid wall thickening, blue discoloration of the colotomy along with patchy black serosal areas (black arrow), and rupture of the suture line (white arrow)

## 1 QUIZ QUESTION: WHAT WENT WRONG?

Methylene blue (MB) is a poor endoscopic tattoo agent occasionally used in clinical practice without standardization in respect of volume and dilution. Currently used agents include India ink (10‐mL sterile vials, diluted 1:50 with normal saline) and Spot (5‐mL sterile vials, ready to use). Methylene blue is less useful with regard to ease of use, safety, and efficacy.^1^ Early reactions especially to high‐volume, low‐dilution MB include mucosal or transmural ischemic ulceration and necrosis resulting in colonic wall abscess, perforation, and peritonitis, as in our patient's case.^2^

## CONFLICT OF INTEREST

None declared.

## AUTHOR CONTRIBUTION

All authors equally accessed the data and contributed to the preparation of the manuscript. BK, BKA, and HA are equally responsible for making and performing treatment decisions. HA reviewed the manuscript for critical intellectual content and had the final approval.

## STATEMENT OF HUMAN AND ANIMAL RIGHTS

The present article does not contain any studies with human or animal subjects performed by any of the authors.

## INFORMED CONSENT

Informed consent was obtained from the patient.
